# The Rasch Analysis of the 25-Item Geriatric Locomotive Function Scale (GLFS-25) in Japanese Older Adults With Musculoskeletal Disorders: Identifying Age-Related Differences in Terms of Item Difficulty and Misfitting Items

**DOI:** 10.7759/cureus.85735

**Published:** 2025-06-10

**Authors:** Masaki Nakano, Tatsunori Ikemoto, Young-Chang P Arai, Nobunori Takahashi, Yukio Nakamura

**Affiliations:** 1 Department of Orthopedic Surgery, Aichi Medical University, Nagakute, JPN; 2 Department of Orthopedics, Aichi Medical University, Nagakute, JPN; 3 Multidisciplinary Pain Center, Aichi Medical University, Nagakute, JPN

**Keywords:** age-related differences, geriatric locomotive function scale, locomotive syndrome, musculoskeletal disorders, rasch analysis

## Abstract

Background and objective

Locomotive syndrome (LS), a condition characterized by diminished mobility due to musculoskeletal disorders (MSDs), is a growing concern among older adults. The 25-item Geriatric Locomotive Function Scale (GLFS-25) is a common tool for LS assessment. However, its reliance on classical test theory (CTT) and the inclusion of non-motor function items raise questions about its accuracy in reflecting the severity of motor dysfunction. This study aimed to evaluate the psychometric properties of the GLFS-25 using Rasch analysis, focusing on variations in item difficulty between young-old (60-74 years) and old-old (75-89 years) individuals with MSDs.

Methods

This cross-sectional study involved 1,000 outpatients (500 young-old and 500 old-old) with MSDs. The participants completed the GLFS-25. Rasch analysis was performed using Winsteps software (Version 5.2.2; www.winsteps.com) to assess item difficulty, individual ability, and item fit. Wright person-item maps were generated to visualize the distribution of item difficulty and individual ability. Infit and outfit mean-square values were used to identify misfitting items.

Results

The mean age of participants was 73.8 ± 6.8 years. Mean GLFS-25 scores were 26.4 ± 22.3 (young-old) and 35.1 ± 23.0 (old-old). Cronbach's alpha exceeded 0.95 in both groups. Significant differences in LS severity proportions were observed between age groups (p<0.001). Wright maps revealed a scarcity of differentiating items among low-scoring individuals, particularly in the young-old group. Items related to dressing, toilet use, and bathing were most differentiating for high-scoring individuals. Neck/upper limb pain and social engagement were identified as misfitting items across both age groups. Back/lower back/buttock pain and social interaction were misfitting in the young-old and old-old groups, respectively.

Conclusions

While the GLFS-25 demonstrated excellent internal consistency, Rasch analysis revealed limitations in its ability to differentiate among individuals with low LS scores, particularly in the young-old group. In addition, several misfitting items were identified, suggesting that some items may not contribute effectively to the measurement of LS.

## Introduction

In 2007, the Japanese Orthopaedic Association (JOA) introduced the concept of locomotive syndrome (LS) to promote musculoskeletal health awareness among older adults. LS is characterized by diminished mobility due to musculoskeletal disorders (MSDs) affecting bones, joints, cartilage, muscles, and nerves: for example, osteoarthritis of the spine and knee osteoarthritis [1,2]. The 25-item Geriatric Locomotive Function Scale (GLFS-25) is a self-administered LS assessment tool [3]. This instrument evaluates pain, paresthesia, motor difficulties, ambulation, and social participation in middle-aged and older individuals. Each item uses a 5-point Likert scale (0-4: no impairment to severe impairment), yielding a total score ranging from 0 to 100 [4]. Studies have demonstrated correlations between the GLFS-25 and reduced gait speed [5], elevated fall risk [6,7], and diminished quality of life in older adults [3,8]. Longitudinal studies have further linked the GLFS-25 to LS incidence [9] and the necessity for long-term care [10,11]. With increasing life expectancy, LS assessment and management have become paramount worldwide [12-15].

However, the GLFS-25 does not directly quantify motor function, encompassing items related to pain, paresthesia, activities of daily living, and anxiety. Consequently, while the instrument assesses LS severity, the total score derived from classical test theory (CTT) may not precisely reflect motor dysfunction severity [16,17]. In a prior study, we proposed the reduction of items in the instrument to enhance its efficacy [18]. However, these reductions have not been newly validated, and the JOA continues to use criteria based on CTT theory. Therefore, in this study, we decided to validate the method by using Rasch analysis.

Rasch analysis, developed by Georg Rasch [19], posits that items within a measurement instrument occupy a unidimensional continuum based on difficulty. This model evaluates the congruence between expected and observed scores. Unidimensionality is assessed via goodness-of-fit statistics and principal component analysis (PCA) of residuals. In other words, even in a population with MSDs, there may be differences in disease severity, and the same measure may not be uniformly quantitative, since the distribution of scores for certain question items may be underestimated or overestimated due to multidimensional question items. In Rasch analysis, the difficulty of each item is expressed in a unique unit called a logit, which normalizes and rates each question item. Rasch analysis enhances measurement quality by weighting individual items according to their contribution to the latent trait, transforming raw scores into continuous data. Furthermore, it identifies misfitting items that are redundant for precise measurement, facilitating their removal. Rasch analysis is increasingly utilized to refine the design and validity of healthcare questionnaires [20-22].

A recent study indicated that two physical performance measures, the two-step test and the stand-up test, exhibited greater sensitivity to aging than the GLFS-25, with the GLFS-25 reference value increasing in individuals aged 75 and older [23]. In light of this, we hypothesized that age differences could result in differences in the appropriate items for LS within the GLFS-25. Therefore, this study investigated GLFS-25 characteristics in Japanese older adults using Rasch analysis, focusing on item difficulty variations between young-old (60-74 years) and old-old (75-89 years) individuals with MSDs.

This article was previously posted to the Research Square preprint server on Feb 13, 2025 (doi.org/10.21203/rs.3.rs-6002959/v1).

## Materials and methods

Study design

This cross-sectional study adhered to the Strengthening the Reporting of Observational Studies in Epidemiology (STROBE) guidelines [24].

Group classification

The World Health Organization classifies individuals aged 65 years and older as elderly, whereas in the United States, this designation is often applied to those aged 60 years and older. In contrast, a recent proposition in Japan designates individuals aged 75 years and older as elderly, underscoring the global variability in this chronological definition. Consequently, for the present investigation, older adult participants were stratified based on age categorizations pertinent to the Japanese context: the 'young-old' (60-74 years) and the 'old-old' (≥75 years).

Sample size

Rasch analysis, an item response theory-based method, benefits from larger sample sizes for improved model fit. Specifically, item difficulty and individual ability estimation accuracy are enhanced with sample sizes of 500 or greater [25]. Given the study's objective to detect intergroup item differences, a sample size of 500 per group was determined to be sufficient.

Study participants

Participants (young-old: n=500; old-old: n=500) were recruited from Aichi Medical University Hospital outpatients between 2018 and 2019. Eligible individuals were invited to complete the GLFS-25 until target sample sizes were achieved for each age stratum. Questionnaires were administered as part of a medical examination to investigate the patient's life obstacles and were self-administered with researcher assistance provided as needed. Inclusion criteria were patients aged 60-89 with MSDs. Exclusion criteria were inability to ambulate independently (with or without assistive devices), blindness, strong hearing loss, and confirmed or suspected dementia. The target sample size (n=1,000) was achieved. Participants were instructed to complete a self-administered questionnaire. If the questions were unclear to the participant, the study physician provided support, explained the purpose of the study comprehensively, and assisted the participant in completing and answering the questionnaire.

The Aichi Medical University Hospital Research Ethics Board approved this study (2019-H136), which was conducted per the principles of the Declaration of Helsinki. The hospital obtained comprehensive consent for the academic use of treatment-related information (e.g., treatment modalities, progress, and other relevant data) from all patients. Study details were accessible to all eligible participants via the hospital's website. Given the retrospective nature of the study, written informed consent was waived. De-identified data were used exclusively for this study unless individual patients opted out.

Outcome measures

GLFS-25 items and response options are presented in the Appendices. Participants responded to each item (Q1-Q25) on a 5-point scale (0-4: better to worse), yielding total scores from 0 to 100. A higher score indicates worse LS status, and scores of ≤6, 7-15, 16-23, and ≥24 denote no-LS, LS-1, LS-2, and LS-3, respectively [4].

Statistical analysis

The Rasch analysis, a probabilistic logistic model that yields logit-based estimations of item difficulty and person ability, was conducted using Winsteps software (Version 5.2.2; www.winsteps.com). This methodology transforms ordinal raw scores into interval-level data by differentially weighting each item's contribution to the underlying latent trait. Initially, the dimensionality of the data was evaluated. Within the Rasch framework, unidimensionality is assessed via PCA of the standardized residuals. A scale is deemed unidimensional if the eigenvalue of the first principal component is below 2.0, and the variance explained by the Rasch dimension exceeds 50% [26]. To visualize the distributions of item difficulty and individual ability, Wright person-item maps were generated for the entire cohort and specific age subgroups. These maps highlight items effective in differentiating individuals with lower and higher levels of the measured trait. Furthermore, such maps were constructed for distinct LS severity classifications.

The Rasch model presupposes unidimensionality and quantifies the contribution of each item to the targeted construct. Logit values possess a probabilistic interpretation: greater logit values correspond to an increased likelihood of endorsing an item or achieving a higher score on an item for individuals exhibiting higher overall ability. Fit statistics, specifically infit and outfit mean-square (MNSQ) values derived from standardized residuals, were scrutinized. Items conforming perfectly to the unidimensional Rasch model exhibit an expected MNSQ value of 1.0. Infit MNSQ is an information-weighted statistic, whereas outfit MNSQ is an unweighted statistic more susceptible to idiosyncratic responses to outlying items. Items were considered to demonstrate acceptable fit if their infit and outfit MNSQ values were ≤1.5 [26]; consequently, items surpassing this criterion for both indices were identified as misfitting. Cronbach's alpha and chi-squared tests for LS severity proportions were performed using R version 4.4.2 [27].

## Results

Participant characteristics

Sixteen participants were excluded from the study for the following reasons: inability to walk independently (n=8), cognitive impairment (n=6), and severe hearing loss (n=2). Table 1 presents the participant characteristics. The mean age of the cohort was 73.8 ± 6.8 years. Mean GLFS-25 scores were 26.4 ± 22.3 (young-old) and 35.1 ± 23.0 (old-old). Cronbach's alpha exceeded 0.95 across groups. LS severity proportions differed significantly between age groups (p<0.001, Table 2).

**Table 1:** Characteristics of the study participants GLFS-25: the 25-item Geriatric Locomotive Function Scale; SD: standard deviation

	All participants (60–89 years: n=1,000)	Young-old (60–74 years: n=500)	Old-old (75–89 years: n=500)
Sex, male/female	385/615	204/296	181/319
Age, years, mean ± SD	73.8 ± 6.8	68.2 ± 4.0	79.4 ± 3.8
GLFS-25, points, mean ± SD	30.7 ± 23.1	26.4 ± 22.3	35.1 ± 23.0
Cronbach's alpha	0.967	0.968	0.964
Raw variance explained by measures, %	62.1	62.3	61.2
Raw unexplained variance, %	37.9	37.7	38.8
Eigenvalue of the 1st contrast	3.16	3.15	3.56
Eigenvalue of the 2nd contrast	3.07	2.77	3.09
Eigenvalue of the 3rd contrast	2.11	2.18	1.91
Eigenvalue of the 4th contrast	1.50	1.50	1.57
Eigenvalue of the 5th contrast	1.28	1.29	1.28

**Table 2:** Proportions of LS severity GLFS-25: the 25-item Geriatric Locomotive Function Scale; LS: locomotive syndrome

	No-LS (GLFS-25, ≤6)	LS-1 (GLFS-25, 7–15)	LS-2 (≤16 GLFS-25, 16–23)	LS-3 (GLFS-25, ≥ 24)
All participants, n (%)	138 (13.8)	191 (19.1)	135 (13.5)	536 (53.6)
Young-old, n (%)	96 (19.2)	112 (22.4)	66 (13.2)	226 (45.2)
Old-old, n (%)	42 (8.4)	79 (15.8)	69 (13.8)	310 (62.0)

Unidementionality

The proportions of variance explained by the Rasch model for all subjects, the young-old group, and the old-old group were 62.1%, 62.3%, and 61.2%, respectively, and the eigenvalues of the first contrast were 3.16, 3.15, and 3.56, respectively (Table 1). This suggests that the questionnaire evaluations for each group may be multidimensional.

Person and item estimates

**Figure 1:**
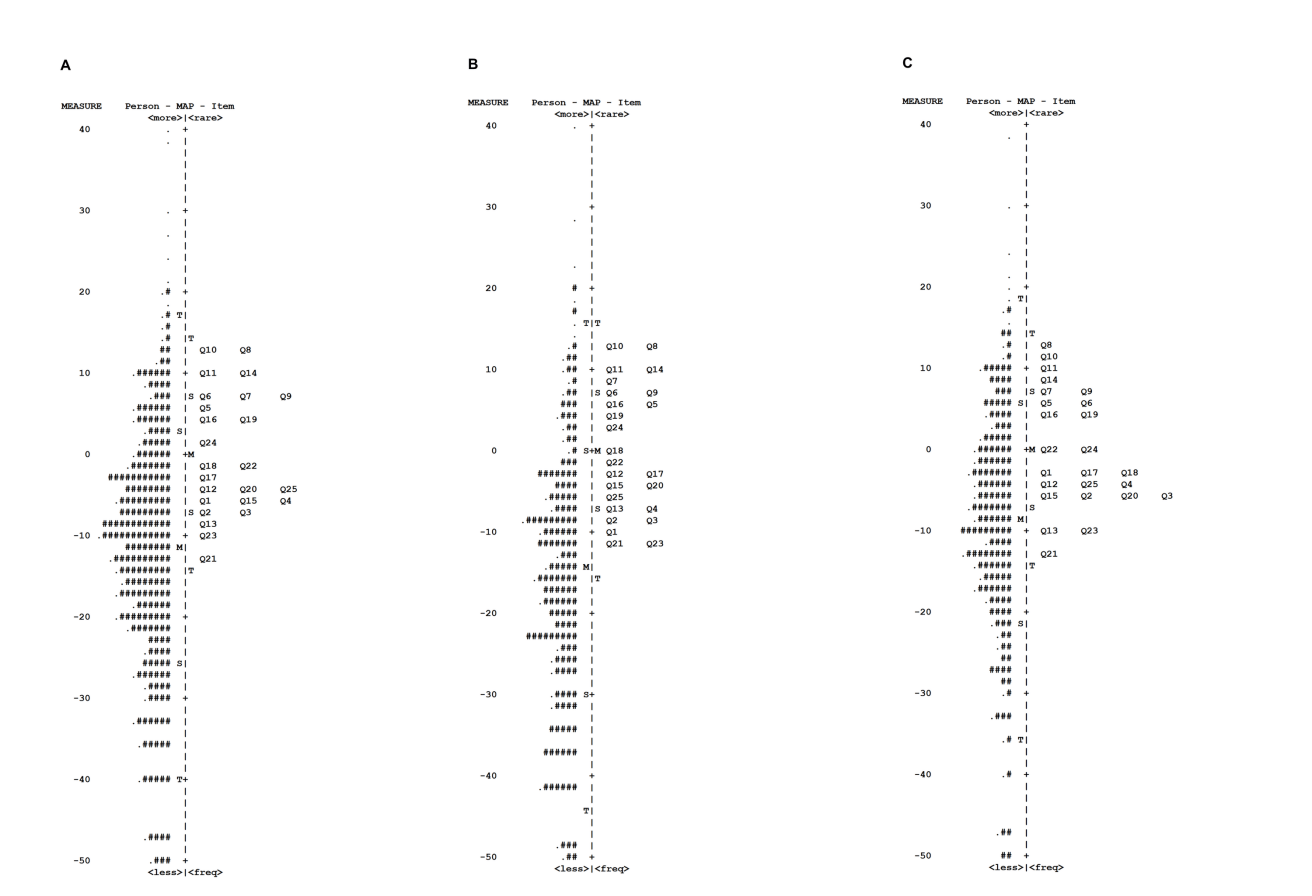
Wright person-item map for the GLFS-25 by age group (A) All participants (60–89 years old). (B) Young-old subgroup (60–74 years old). (C) Old-old subgroup (75–89 years old) GLFS-25: the 25-item Geriatric Locomotive Function Scale

**Figure 2:**
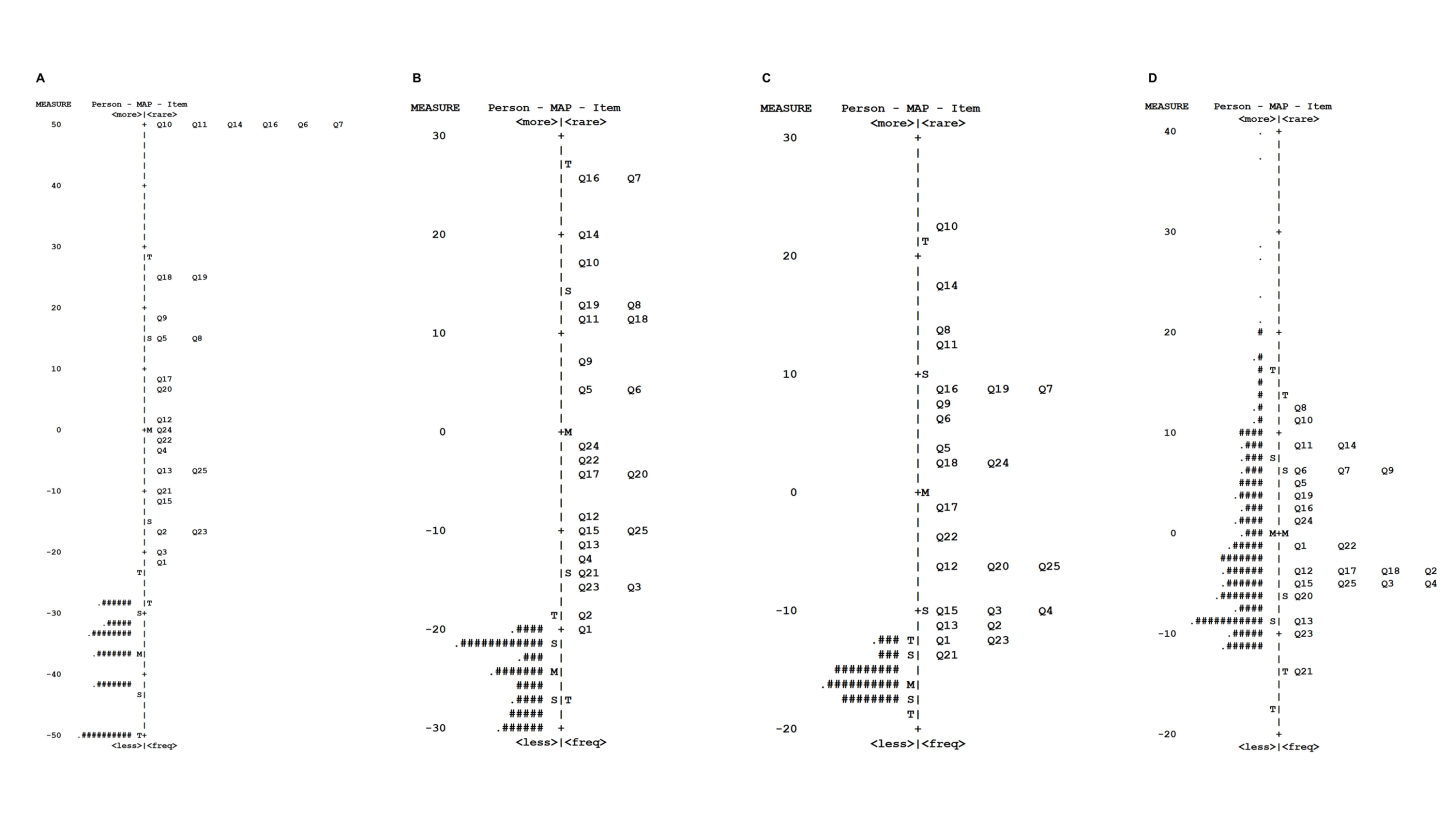
Wright person-item map for the GLFS-25 by LS severity (A) No-LS (≤6 points). (B) LS-1 (7–15 points). (C) LS-2 (16–23 points). (D) LS-3 (≥24 points) GLFS-25: the 25-item Geriatric Locomotive Function Scale; LS: locomotive syndrome

Wright maps (Figure 1) depict item difficulty and individual ability distributions by age group. Items such as Q8 (dressing), Q10 (toilet use), and Q11 (bathing) were effectively differentiating items among high-scoring individuals across groups. Conversely, differentiating items for low-scoring participants, particularly in the young-old group, were scarce. Items such as Q13 (brisk walking), Q21 (sports participation), and Q23 (social engagement), along with pain/paresthesia questions (Q1-Q4), were differentiating items among mid-range scorers. Stratified by LS severity, while the GLFS-25 effectively differentiated the LS-3 group, appropriate items were deficient within the no-LS, LS-1, and LS-2 groups (Figure 2).

Item difficulty logit values are presented in Table 3. Q8, Q10, Q11, and Q14 exhibited high logit values, while Q21 and Q23 exhibited low logit values across age groups.

**Table 3:** Item difficulty measures SE: standard error

	All participants		Young-old		Old-old	
	Measure	SE	Measure	SE	Measure	SE
	(logits)		(logits)		(logits)	
Q1	-6.00	0.37	-9.34	0.54	-3.24	0.51
Q2	-6.71	0.37	-8.55	0.54	-5.31	0.51
Q3	-7.37	0.37	-8.90	0.54	-6.26	0.51
Q4	-5.75	0.37	-7.26	0.55	-4.63	0.51
Q5	5.35	0.43	5.49	0.67	5.34	0.57
Q6	6.53	0.44	7.20	0.69	6.17	0.58
Q7	7.65	0.45	8.13	0.71	7.46	0.60
Q8	12.29	0.51	12.24	0.79	12.56	0.67
Q9	7.17	0.45	7.29	0.69	7.21	0.59
Q10	12.39	0.51	13.19	0.81	12.06	0.66
Q11	9.34	0.47	9.37	0.73	9.50	0.62
Q12	-3.69	0.38	-2.68	0.58	-4.61	0.51
Q13	-8.70	0.37	-7.64	0.54	-9.89	0.51
Q14	9.63	0.48	10.46	0.75	9.23	0.62
Q15	-5.60	0.37	-4.81	0.56	-6.41	0.51
Q16	4.67	0.43	5.62	0.67	4.06	0.56
Q17	-2.53	0.38	-2.34	0.58	-2.77	0.52
Q18	-1.51	0.39	-.33	0.60	-2.47	0.52
Q19	4.57	0.43	4.83	0.66	4.47	0.56
Q20	-4.76	0.37	-4.52	0.56	-5.10	0.51
Q21	-12.18	0.36	-11.70	0.53	-13.01	0.51
Q22	-1.01	0.39	-1.70	0.58	-0.52	0.53
Q23	-10.46	0.36	-11.33	0.53	-10.00	0.51
Q24	1.61	0.40	2.91	0.63	0.66	0.53
Q25	-4.93	0.37	-5.62	0.55	-4.50	0.51

**Figure 3:**
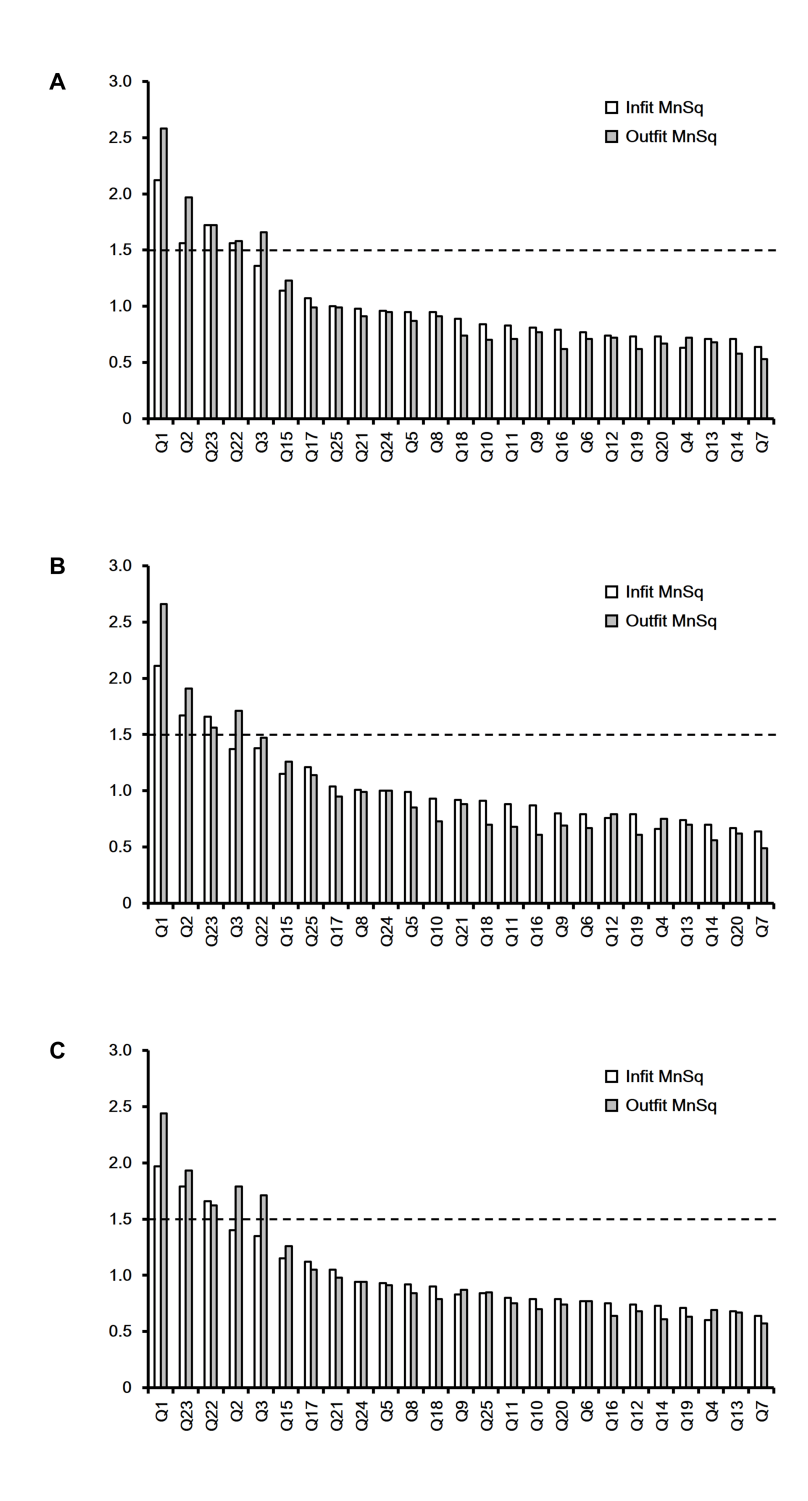
Infit and outfit MNSQ values for the GLFS-25 by age group (A) All participants (60–89 years old). (B) Young-old subgroup (60–74 years old). (C) Old-old subgroup (75–89 years old) The dashed line indicates the MNSQ value of 1.5 GLFS-25: the 25-item Geriatric Locomotive Function Scale; MNSQ: mean-square

Mean-square values (Figure 3) revealed Q1 (neck/upper limb pain) and Q23 (social engagement) as misfitting (infit and outfit ≥1.5) across groups. Additionally, Q2 (back/lower back/buttock pain) and Q22 (social interaction) were misfitting in the young-old and old-old groups, respectively. Q7 (indoor ambulation) demonstrated the highest sensitivity across groups.

## Discussion

This is the first study to investigate GLFS-25 characteristics and validity among Japanese older adults using the Rasch analysis. Cronbach's alpha indicated excellent internal consistency across groups. While the GLFS-25 reflected LS-3 severity, differentiating items for low-scoring individuals, especially in the young-old group, were limited. Furthermore, misfitting items varied between age groups.

As LS is an epidemiological construct, GLFS-25 assessment, alongside physical function tests, remains an official diagnostic criterion [2]. However, pain and psychological status may be overestimated in younger individuals due to greater social roles and burdens [28], even with adequate muscle strength and balance. Conversely, reduced social roles and activities in older individuals may result in lower GLFS-25 scores despite motor impairment. Therefore, items and severity levels contributing to locomotive difficulties may vary across age groups. CTT, the most prevalent theory, employs simple mathematical analysis. GLFS-25-based LS assessment also utilizes this theory. However, our findings suggest the validity of utilizing a single composite score for assessing LS severity across all individuals is questionable. A study found that older patients (aged 65 and over) reported better scores on certain items, suggesting better outcomes in specific contexts [29].

Wright person-item maps demonstrated that the items were geared towards middle to high scores on the questionnaire across groups. The median score for all participants was 26.0, suggesting a lack of appropriate questions to differentiate between LS-1 and LS-2. Indeed, suitable items were deficient within the no-LS, LS-1, and LS-2 groups, particularly in the young-old group, highlighting the need for improved early LS assessment and management.

Misfitting items introduce noise. Analysis revealed Q1 (neck/upper limb pain), Q2 (back/lower back/buttock pain), Q22 (social interaction), and Q23 (social engagement) as misfitting. Of these, Q2 and Q22 were characteristic of the young-old and old-old groups, respectively. These findings suggest that items related to body pain/paresthesia and social activities tend to misfit, with potential intergroup variations. The reasons for these items to be misfit cannot be analogized from this analysis, but in general terms, there may be individual variations in pain perception due to aging and differences in family environment and lifestyle during the aging period. Consistent with our prior study employing confirmatory factor analysis, item refinement may be necessary. Beyond highlighting patient-reported outcome measure (PROM) limitations, our results suggest considering the target population and relevant items when establishing PROM [30].

Limitations

This study has certain limitations, such as its single-institution recruitment, which potentially introduces sampling bias and limits generalizability. Firstly, inconsistency in researcher assistance during questionnaire completion could have led to biased outcome measurements. Secondly, although two functional assessments (the two-step test and the stand-up test) were used alongside the GLFS-25 to evaluate LS, this study focused on evaluating methods for psychological characteristics; thus, the relevance of these functional tests was not addressed. Future research should examine the correlation between GLFS-25 items and these functional measures. Thirdly, comorbidities and quality of life were not assessed. Additionally, the exclusion of non-ambulatory elderly individuals meant that potentially relevant patient backgrounds and social environments were not considered. Elderly individuals often have multiple comorbidities and varying psychosocial factors influencing LS. Fourthly, IRT models include 1-parameter logistic (1PL), 2-parameter logistic (2PL), and 3-parameter logistic (3PL) models. This study employed only the 1PL model to identify suitable or misfit items for the target groups. More flexible methodologies, such as 2PL or multidimensional models, warrant consideration in future research. Further studies are also needed to address concurrent validation.

## Conclusions

The study utilized the Rasch analysis to evaluate the GLFS-25 in Japanese older adults with MSDs. It revealed that while the GLFS-25 effectively identified severe locomotive syndrome, it struggled to differentiate between milder LS stages, especially in young-old individuals. The analysis also identified misfitting items, such as those related to pain and social engagement, indicating that these items might not accurately reflect motor dysfunction severity. The research highlights the necessity for refining the GLFS-25 to improve its accuracy in assessing varying levels of LS across different age groups.

## Figures and Tables

**Figure 4:**
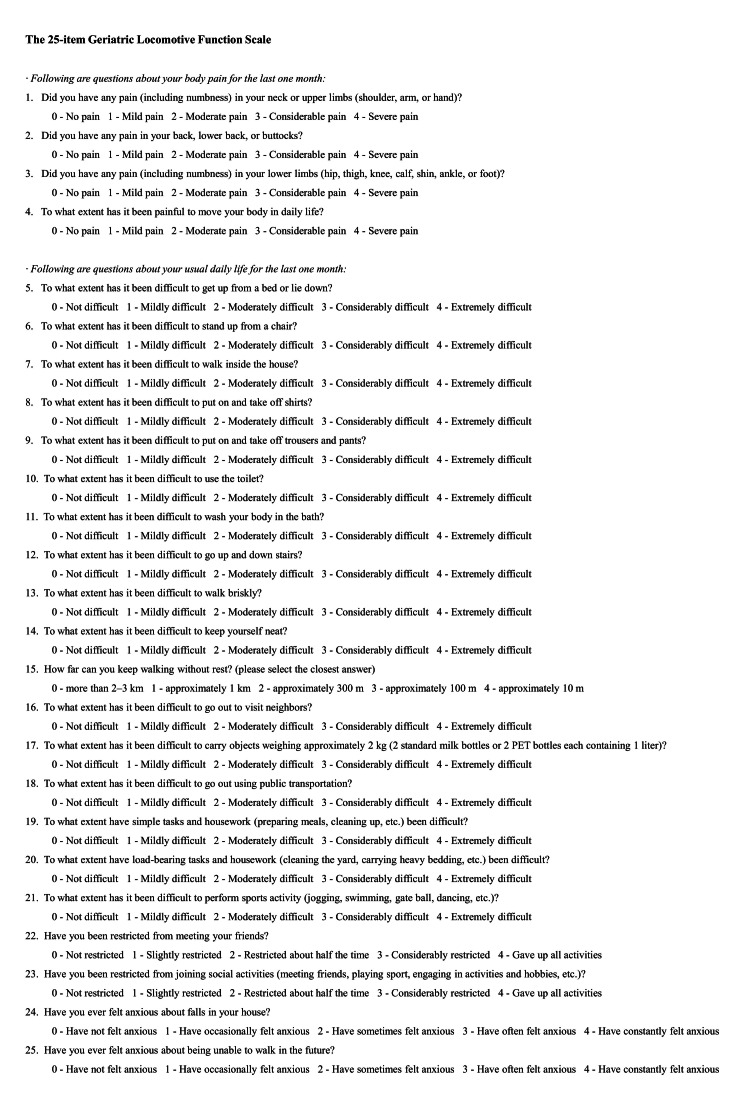
The 25-item Geriatric Locomotive Function Scale
